# Effects of illuminance and correlated color temperature of indoor light on emotion perception

**DOI:** 10.1038/s41598-021-93523-y

**Published:** 2021-07-12

**Authors:** Yun Li, Taotao Ru, Qingwei Chen, Liu Qian, Xianghang Luo, Guofu Zhou

**Affiliations:** 1 School of Psychology, South China Normal University, Guangzhou, 510631 China; 2 Lab of Light and Physio-psychological Health, National Center for International Research on Green Optoelectronics, South China Normal University, Guangzhou, 510006 China; 3 Guangdong Provincial Key Laboratory of Optical Information Materials and Technology & Institute of Electronic Paper Displays, South China Academy of Advanced Optoelectronics, South China Normal University, Guangzhou, 510006 China

**Keywords:** Human behaviour, Psychology and behaviour

## Abstract

The acute non-image forming (NIF) effects of daytime light on momentary mood had been-although not always-established in the current literature. It still remains largely unknown whether short-time light exposure would modulate emotion perception in healthy adults. The current study (N = 48) was conducted to explore the effects of illuminance (100 lx vs. 1000 lx at eye level) and correlated color temperature (CCT, 2700 K vs. 6500 K) on explicit and implicit emotion perception that was assessed with emotional face judgment task and emotional oddball task respectively. Results showed that lower CCT significantly decreased negative response bias in the face judgment task, with labeling ambiguous faces less fearful under 2700 K vs. 6500 K condition. Moreover, participants responded slightly faster for emotional pictures under 6500 K vs. 2700 K condition, but no significant effect of illuminance or CCT on negativity bias was revealed in the emotional oddball task. These findings highlighted the differential role of illuminance and CCT in regulating instant emotion perception and suggested a task-dependent moderation of light spectrum on negativity bias.

## Introduction

Light therapy has been established as an effective antidepressant treatment for patients with seasonal and nonseasonal affective disorders^1–3^. Several meta-analyses of randomized, placebo-controlled trials confirmed that light therapy was more effective than a placebo in patients with affective disorders^3–5^. Light can induce indirect regulation of mood by modulating circadian rhythms and sleep^6^. It was well documented that aberrant light stimulation can cause problems with circadian rhythms and sleep, which can result in the development of mood disorders^7–9^. In addition to the indirect pathway, light can directly regulate mood by activating brain regions (i.e., the medial amygdala and lateral habenula) involving emotional processing, which was not necessarily mediated by the suprachiasmatic nucleus (SCN). The main driver for these so-called non-image-forming effects (NIF) effects of light is the intrinsically photosensitive retinal ganglion cells (ipRGCs) containing the photopigment melanopsin, with peak sensitivity in the blue part of the electromagnetic spectrum^10–12^.

To date, we know little about how light effectively works in the treatment of affective disorders. Negativity bias was identified as playing an important role in developing and maintaining major depressive disorder (MDD)^13,14^, which describes the phenomenon whereby individuals selectively pay more attention to negative stimuli than neutral and positive stimuli^15,16^ or are inclined to judge an ambiguous stimulus as negative^17,18^. The above findings suggested that individuals who experience symptoms of depression show greater negative bias than people without such symptoms^13,15–18^. Negativity bias, in turn, could predict both the development of severe symptoms in the future and the development of a persistent depressive episode among people with depression^19–24^. However, whether specific artificial light exposure would moderate the negativity bias remains largely unknown.

In addition to the antidepressant effects of long-term light exposure, the acute non-image forming effects (NIF) of short-term light exposure on subjective mood were reported in multiple empirical studies^25–30^. The current literature has-although not always-demonstrated the benefits of manipulating light intensity for a short duration (30–120 min) on momentary mood during daytime^25–27,31^. For instance, one study by Smolders and de Kort^26^ revealed that participants felt significantly happier in 1000 lx vs. 200 lx conditions (at eye level) after 30 min of light exposure, though participants' feeling of sadness remained unaffected with light level. Another recent study by Ru and colleagues reported that participants' negative mood assessed with Positive Affect and Negative Affect Schedule (PANAS) significantly decreased after 50 min of 1000 lx vs. 100 lx light during daytime working hours^25^. By contrast, several studies revealed null effects of light intensity on subjective affective state^28,32–34^. This case was also true for the acute effect of manipulating light's spectrum on subjective indicators of mood. Note that the correlated color temperature (CCT) of polychromatic light generally increases when it contains more power in the blue part of the spectrum. The findings of these studies have reported either benifitial^29,35,36^ or impaired^30^ and null^32,37,38^ effects of correlated color temperature (CCT) on subjective mood. For instance, Hawes et al.^35^ reported that participants' depression scores, as assessed by the profile of mood states (POMS), decreased with higher color temperatures (5448 K and 6029 K vs. 3345 K; 90 min), suggesting that mood can be enhanced by higher CCT. In contrast, Smolders and de Kort^30^ found that subjects felt less happy and sadder as assessed with a single 4-point item under high vs. low CCT (6000 K vs. 2700 K; 90 min) conditions.

Furthermore, quite a few empirical studies were conducted in an effort to investigate the interaction effects of illuminance and spectrum of light on and subjective mood^25,39–41^. Lan et al.^42^ found that participants reported more positive mood in the standard warm light (300 lx, 3000 K) and bright cool light (2000 lx, 6000 K) condition than in the bright warm light (2000 lx, 3000 K), while Ru et al.^25^ did not reveal statistically significant interaction effect between light level and CCT on subjective mood as assessed with PANAS. The above inconsistent findings could partly be explained by the differences in light properties and time factor, while the differential subjective indicators of mood employed in these studies may also be the factor leading to the variability of the light's regulation of mood.

To date, a few studies investigated the effects of illuminance or/and spectrum of light on emotional processing^43–48^. For instance, Fotios et al.^43^ reported that the intensity of ambient light had significant effects on expression recognition, such that the probability of correctly identifying emotions conveyed by facial expression was increased with the increased light level. Whereas one subsequent study by Fotios et al.^45^ revealed that the spectrum of lamp light (2000 K vs. 4000 K) did not significantly affect the ability to recognize facial expressions. A recent study by Yoshiike et al.^48^ reported that a 15-min bright light (9000 lx) facilitated fear extinction and antagonized fear acquisition after 24 h and regulated the hemodynamic response of the prefrontal cortex involving in the top-down regulation of fear. These findings suggested that short-term light exposure had the potential to modulate the perception of emotional stimulus. Yet, the acute effects of short-term light exposure on the processing of different valences (i.e., positive, neutral, and negative) of emotional stimuli, especially the regulation of negativity bias, were scarcely investigated. As we mentioned above, a reduction in negative biases in interpretation and appraisal of intrusions was usually associated with a reduction in depressive symptoms over the same period^49,50^. It is of great practical significance to explore the regulation of ambient light on this specific emotional processing.

The potential mechanism by which light regulates emotional processing still remains unclear. There were evidences that ipRGCs project to multiple emotional relevant brain regions, among which are cortical areas involved in the top-down regulation of emotional attention (such as the dorsolateral prefrontal cortex, intraparietal sulcus, and superior parietal lobule) and subcortical areas related to the bottom-up reorientation of attention (the right insula, the anterior cingulate cortex, and the superior temporal sulcus)^51–55^. These findings may provide an insight that light exposure would moderate both the implicit (Top-down) and explicit (Bottom-up) processing to emotional stimulus. Also, given that the subjective mood may interact with light, thus modulating objective emotional processing^56,57^, it is necessary to explore the specific effects of light on instant emotional processing with careful monitoring subtle influence from emotional states.

Thus, the current study was conducted in an effort to investigate the influence of light level and spectrum of indoor light on explicit emotional perception as assessed with facial expression judgment task and implicit emotional perception as assessed with the emotional oddball task. Forty-eight healthy participants were assigned to perform both emotional judgment task and emotional detection task under two illuminance levels (100 lx and 1000 lx at eye level) and two CCT levels (2700 K and 6500 K). To dissociate the specific light's effects on emotional perception from subjective mood, we monitored the subjective momentary mood and sleepiness prior to the laboratory study and further put them as covariates. To the best of our knowledge, the current study is the first time to explore the influence of indoor light on both explicit and implicit emotional perception with careful controlling of subtle influence from emotional states. Our study may provide further insights into indoor lighting design or provide guidance on light therapy.

## Methods

### Experimental design

A 2 (CCT level: 2700 K vs. 6500 K, between-subjects) × 2 (Illuminance: 100 lx vs. 1000 lx at eye level, within-subjects) mixed-model design was employed in the current study. Participants came to the laboratory on two separate days with an interval of at least 2 days. The laboratory study was conducted from May to August 2019. All participants were free to register either in the morning session (8:30–12:30) or in the afternoon session (14:30–17:30), and the local clock time remained the same on the two experiment days. The order of illuminance condition was counterbalanced between participants.

### Participants

A power analysis was conducted before the formal experiment using G*power^58^; each group needs 24 participants to achieve 95% power when the effect size partial η^2^ was hypothesized to be 0.14^59^. Forty-eight healthy young volunteers (mean age = 20.12 years, SD = 1.76 years, 37 females) were recruited from local universities via advertisements; 24 participants (19 females) were assigned into 2700 K condition, while the other half (18 females) received the light at 6500 K. All the participants had a normal or corrected-to-normal vision and were right-handed. None of them were extreme chronotypes according to the Morningness-Eveningness Questionnaire^60^. Besides, they had no indication of emotional disturbance based on the Beck Depression Inventory-II^61^ and the Beck Anxiety Inventory^62^. The study was approved by the Human Research Ethics Committee for Experiment involving Human Species at Local University, and all research activities have adhered to the principles of the Declaration of Helsinki. All participants gave their written informed consent before starting the laboratory study and got payment for participation.

### Experimental setting

**Figure 1:**
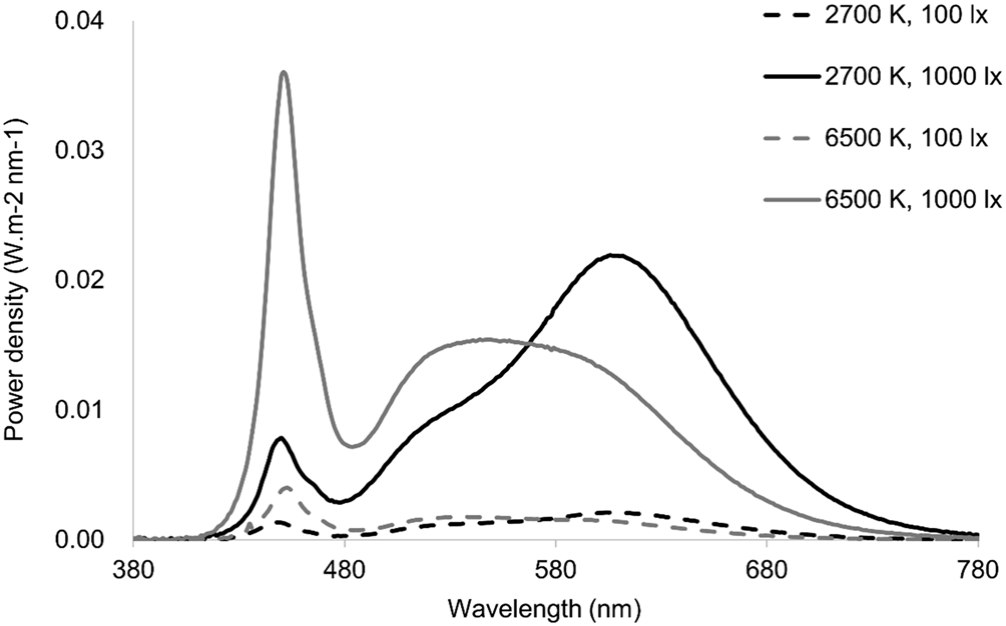
Spectral power distribution was measured at eye level in the four lighting conditions.

The experiment was conducted in a room furnished as a simulated office (4.1 m by 3.3 m by 2.9 m). There were four white workstations separated by white panels; each workstation included a pure white desk (1.2 m by 0.8 m) and a black chair, and on the desk was a white All-in-One PC (Lenovo C260, 19.5 inches) with a pair of headphones, a keyboard, and a mouse. Six recessed Philips Savio luminaires were mounted on the ceiling of the room. The illuminance level, spectral power distribution (SPD), and color-rendering index (CRI) were measured at eye level by a calibrated spectroradiometer (JETI Specbos1201) aimed at the screen in the gaze direction of participants (Table 1 and Fig. 1). The CRI at 6500 K was Ra = 84, and the CRI at 2700 K was Ra = 86.

**Table 1:** Spectrally weighted α-Optic lux level at eye levels for each lighting condition based on Lucas et al.^91^.

	λmax	α-Optic lux value				
		(4000 K, 200 lx)	(2700 K, 100 lx)	(2700 K, 1000 lx)	(6500 K, 100 lx)	(6500 K, 1000 lx)
Lux		193	103	1016	111	1000
CCT		4034	3094	2710	6634	6571
S-cone	419	127	44	269	114	1031
Melanopsin	480	139	54	446	108	979
Rod	496	151	65	564	110	994
M-cone	530	175	87	820	111	1004
L-cone	558	189	102	1010	107	970

### Measures

#### Subjective mood and sleepiness

The subjective mood was measured with two items ("happy" and "sad" respectively) selected from the activation-deactivation adjective checklist^63^; each item was rated on a five-point Likert scale, ranging from 1 = definitely not to 5 = definitely. Subjective sleepiness was assessed with the Karolinska Sleepiness Scale (KSS)^64^, ranging from 1 = extremely alert to 9 = extremely sleepy.

#### Light sensitivity and evaluation of experimental light

Light sensitivity was measured with three items adopted from Smolders et al.^28^. At the end of the experiment, all participants were asked to evaluate the experimental light (pleasantness, comfort, disturb, warmth, preference, brightness, excitement) using seven 5-point Likert-scale items adopted from Flynn^65^. Additionally, two 5-point Likert scale items adopted from Smolders and de Kort were used to probe participants' beliefs about the current lighting effects on work performance and mood^25^.

### Experimental tasks

#### Emotional face judgment task

**Figure 2:**
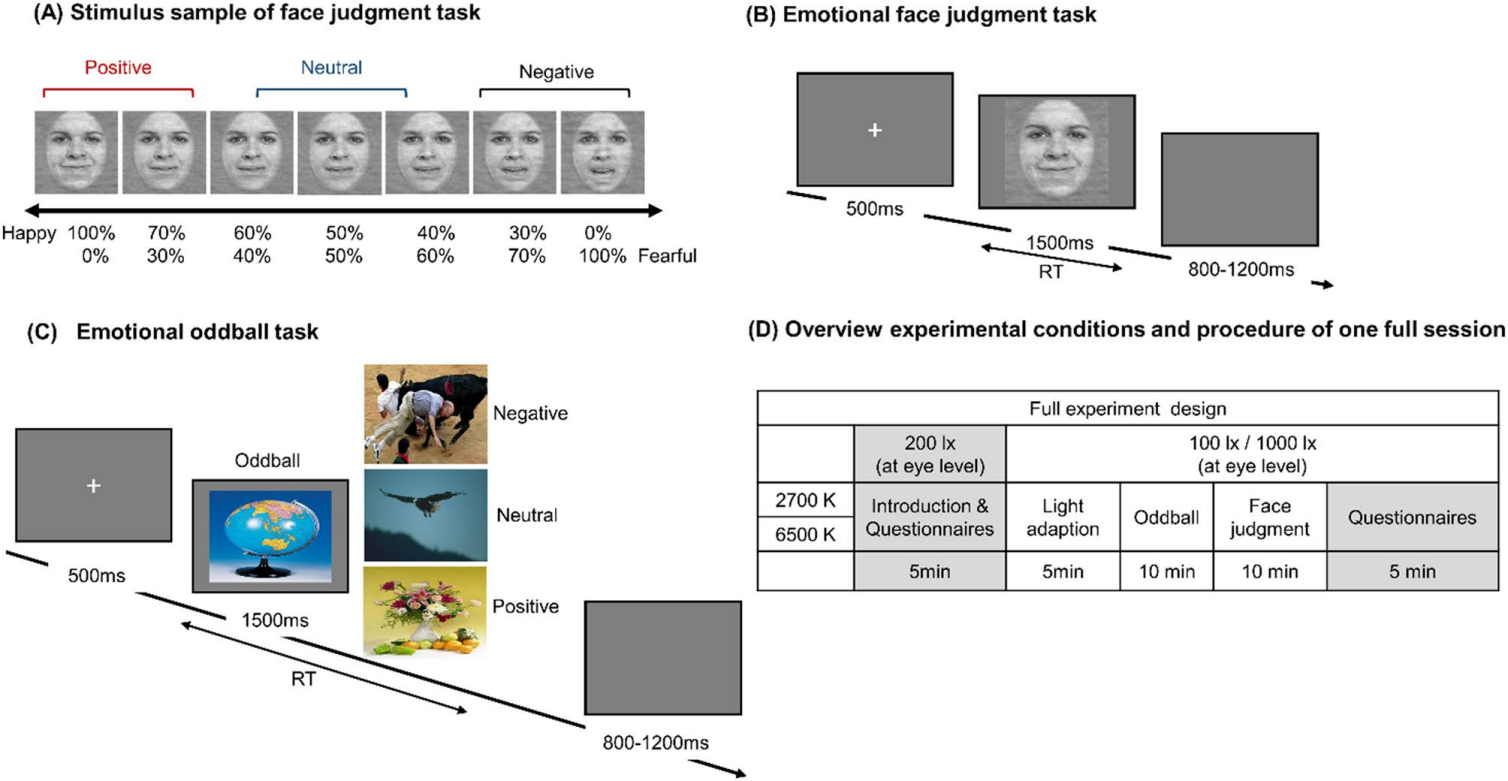
(**A**) Stimulus sample of face judgment task: female, facial expression ranges from 100% happy /0% fearful to 0% happy /100% fearful, all morphs were created by customized scripts in MATLAB R2017a (MathWorks Inc., Natick, MA, USA). (**B**) Emotional face judgment task. A face was presented after a fixation cross, and then participants were required to judge whether the emotion conveyed by the face is "fearful" or "happy" within 1.5 s. (**C**) Emotional oddball task. A picture was presented after a fixation cross, and participants were required to answer whether it is a standard stimulus or an oddball stimulus within 1.5 s immediately. (**D**) Overview of experimental condition and schematic representation of one full session. Note that the task order was the same on two experiment days and counterbalanced between the participants.

The emotional face judgment task was used to assess the explicit perception of emotional stimuli, in which all the faces were on a morphed expression continuum between exemplars of fearful and happy expressions^66^. Two female faces and two men faces were selected from an international database named STOIC database^67^. The unambiguous exemplars of fearful and happy expressions were selected for each individual, and pixel value and location were interpolated between fearful exemplar faces and happy exemplar faces to generate the morphed expression continua for this experiment. Five levels of fear-happy morphs, ranging from 30% fear/70% happy to 70% fear/30% happy in steps of 10%, were created by customized scripts in MATLAB R2017a (MathWorks Inc., Natick, MA, USA), and low-level image properties were equalized by the SHINE toolbox^68^ in MATLAB. At last, seven levels of fear–happy morphs were included, ranging from 0% fear/100% happy to 100% fear/0% happy in steps of 10%, so the faces include seven levels of fearful ratio: 0%, 30%, 40%, 50%, 60%, 70%, 100%. According to the degree of fear, these seven different levels were further divided into three levels: positive, neutral, negative (Fig. 2A). During this task, participants were asked to judge the facial expressions (happy or fearful) presented on the screen as quickly and accurately as possible by pressing one of two labeled response keys on the keyboard. The response hand for happy and fearful faces was balanced between participants. There were two blocks for this task, with each block including 140 trails, and each face was presented for 1500 ms with a random interval of 800 to 1200 ms (Fig. 2B). The reaction time for the three kinds of emotional faces and the selection rate for fearful faces in the seven levels of emotional faces were separately investigated.

#### Emotional oddball task

The emotional oddball task was used to assess the implicit perception of emotional stimulus. A neutral picture (valence = 5.59, arousal = 4.40) was selected as the standard stimulus, whereas the other three kinds of stimuli with a different emotional valance (negative, neutral, and positive) were selected as the oddball stimuli. All the pictures were selected from the Chinese Affective Picture System (CAPS)^69^. The standard stimulus was presented with a probability of 70%, and the oddball stimuli were presented with a probability of 30% (10% for each Valence)^70^. The valence level (Negative, M = 2.05, SD = 0.16; Neutral, M = 5.68, SD = 0.17; Positive, M = 6.77, SD = 0.16) of three kinds of oddball stimulus were evaluated before the main task with significant difference across them (F (2,87) = 602.37, *p* < 0.001), while the arousal level (Negative, M = 5.69, SD = 0.57; Neutral, M = 5.69, SD = 0.75; Positive, M = 5.71, SD = 0.16) were not different significantly (F (2,87) = 0.18, *p* = 0.84). Two blocks were included in this task, with each block consisting of 150 trails. Each stimulus was presented for 1500 ms with a random interval of 800 to 1200 ms (Fig. 2C). Participants were required to respond to the oddball or standard stimulus by pressing one of two labeled response keys as quickly as possible. The response hand for the standard and the oddball stimulus was balanced between participants. The average accuracy and reaction time of the three kinds of emotional pictures were separately recorded for the analyses.

### Experimental procedure

After participants arrived in the laboratory, they were instructed to complete a set of questionnaires with questions about their sleep offset, sleep duration, sleep quality of the preceding night, light sensitivity, how long they spent outdoors and whether they had consumed coffee and/or other drinks containing alcohol or caffeine before they arrived at the laboratory. Baseline sleepiness and mood were assessed before the light manipulation. The experiment always started with a 5-min light adaption after the experimental light was turned on, in which participants were free to read books containing no emotion priming contexts. Afterward, two computerized tasks were programmed with counterbalanced orders, and the order of the task remained consistent for each participant on the two experiment days. At the end of the task session, subjective mood and sleepiness were repeatedly measured, and the subjective evaluation of the experiment light was assessed. A schematic representation of one full experimental session is depicted in Fig. 2D.

### Statistical analyses

Before further statistical analyses were performed, responses on error trials (omissions, false starts, and trials following incorrect responses) and outliers (M ± 3SD; per participant and per dependent variable per session) were removed. Due to the hierarchical structure of the data, linear mixed model (LMM) analyses were conducted to test the effects of Illuminance and CCT on all dependent variables. Participants and the experimental session (nested within participant) were added as random intercepts to cluster the data per participant and per experimental session in all models. Firstly, a preparatory LMM analysis was performed with sleep duration (self-reported hours), sleep quality (GSQS score) of the night before the experiment days, or light sensitivity as the response variable; the models for each of the different dependent variables further included CCT and Illuminance level and their interactions as fixed factors. Secondly, further LMM analyses were performed with the subjective sleepiness and mood before (baseline) and after light manipulation and evaluation of lighting conditions as dependent variables; in those models, CCT and Illuminance and their interaction were added as fixed factors. Confounding variables with significant differences at baseline, baseline subjective mood, and alertness, lighting evaluation variables of post-experiment with a significant difference across conditions were used as control variables in future LMM analysis on emotional processing.

For the face judgment task, an LMM model was built with CCT, Illuminance, Fearful levels of faces or Valence, and their interactions as fixed factors, whereas the percentage of choosing fearful or reaction time work as response variables respectively. For the oddball task, LMM models were built with CCT, Illuminance, Valence, and their interactions as fixed factors, whereas the response accuracy or reaction time as response variables, respectively. Post hoc contrasts were performed to investigate the nature of all significant interaction effects with Bonferroni corrected. Adjusted R-Squared was given for the total mixed model at level 1, i.e., within sessions. This measure is the proportion of reduction in the variance of residuals; note that this measure can also have negative values^71^.

## Results

### Confounding variables: sleep quality, sleep duration, and light sensitivity

Surprisingly, our results revealed a significant main effect of Illuminance on light sensitivity (F (1,48) = 6.08, *p* = 0.02, R^2pseudo^ = − 0.10), the participants reported higher light sensitivity before receiving the high light level manipulation. In addition, a main effect of CCT was found on sleep duration (F (1,48) = 5.81, *p* = 0.02, R^2pseudo^ = 0.05), with shorter total sleep time in the 6500 K condition (8.17 ± 0.16) than in the 2700 K condition (7.65 ± 0.15). Results showed that there were no main effects of CCT or Illuminance on sleep quality, neither the interaction effects of CCT and Illuminance on these three variables (all *p* > 0.01) (Table 2). Thus, sleep duration and light sensitivity were both taken as covariates in further analysis.

**Table 2:** Descriptive statistics results for confounding variables and baseline measures.

	2700 K		6500 K		Statistics									
	100 lx	1000 lx	100 lx	1000 lx	CCT			Illuminance			CCT*illuminance			R^2^
	EMM (SE)	EMM (SE)	EMM (SE)	EMM (SE)	F	*df*	*p*	F	*df*	*p*	F	*df*	*p*	
Sleep duration	7.67 (0.19)	7.62 (0.19)	7.98 (0.19)	8.37 (0.19)	5.81	(1,48)	**0.02**	1.12	(1,48)	0.30	1.86	(1,48)	0.18	0.05
Sleep quality	1.54 (0.54)	2.79 (0.54)	2.50 (0.54)	2.96 (0.54)	0.87	(1,48)	0.36	3.26	(1,48)	0.08	0.70	(1,48)	0.41	0.10
Light sensitivity	7.67 (0.40)	7.13 (0.40)	7.75 (0.40)	7.38 (0.40)	0.10	(1,48)	0.76	6.08	(1,48)	**0.02**	0.20	(1,48)	0.66	− 0.10
Sleepiness (KSS)	2.96 (0.25)	2.88 (0.25)	3.17 (0.25)	2.83 (0.25)	0.08	(1,48)	0.78	1.45	(1,48)	0.24	0.52	(1,48)	0.47	0.05
Happy	3.67 (0.14)	3.63 (0.14)	3.63 (0.14)	3.71 (0.14)	0.02	(1,48)	0.90	0.30	(1,48)	0.90	0.27	(1,48)	0.60	0.01
Sad	2.00 (0.17)	1.71 (0.17)	1.83 (0.17)	1.67 (0.17)	0.26	(1,48)	0.61	2.73	(1,48)	0.11	0.20	(1,48)	0.65	0.05

*EMM* estimated marginal means, *SE* standard errors.

A significant difference is indicated by bold labels.

Effect sizes (pseudo R^2^-values (R^2pseudo^)) indicates the proportion (percentage) of the reduction in the variance of residuals from the null model to the final (full) model at level two; it is worth mentioning that this measure can also have negative values^71^.

### Baseline comparison: sleepiness and mood

For the baseline rating, all the LMM analyses showed no significant main effects or interaction effects of CCT and Illuminance on baseline sleepiness, positive mood, and negative mood (all *p* values > 0.05) (Table 2).

### Effects of lighting on emotion perception

#### Emotional face judgment task

LMM analysis for the percentage of choosing fearful in the face judgment task revealed a significant main effect of CCT (F (1,52) = 4.78, *p* = 0.03); participants were less inclined to make fearful decisions under 2700 K (EMM = 0.49, SE = 0.02) versus 6500 K condition (EMM = 0.55, SE = 0.02, *p* = 0.03). The interaction effect between CCT and fearful levels was also significant (F(6,570) = 2.09, *p* = 0.05, R^2pseudo^ = 0.92). The post-hoc analysis revealed that participants selectively showing significantly lower percentage of choosing fearful for the 30%, 40%, 60% and 70% fearful levels (all *p values* < 0.05) under 2700 K vs. 6500 K condition, but not for 0% and 100% fearful﻿ level.

To further investigate participants’ sensitivity of emotional judgment in two CCT conditions, a logistic function was fitted to obtain smooth psychometric curves under two CCT conditions. The Eq. (1) is as follows:

1 $$P\left( x \right) = \frac{{P_{{inf}} }}{{1 + e^{{ - \alpha \left( {\chi - \chi_{{half}} } \right)}} }}$$

*P* is the percentage of trials judging faces as fear, χ is the morph level, *P*_inf_ is the value when χ approaches infinity (the curve's maximum value), χ_half_ is the symmetric inflection point (the curve's midpoint), and α is the steepness of the curve. *P*_inf_, χ_half_, and α were fitted from the observed data (*P* and χ).

**Figure 3:**
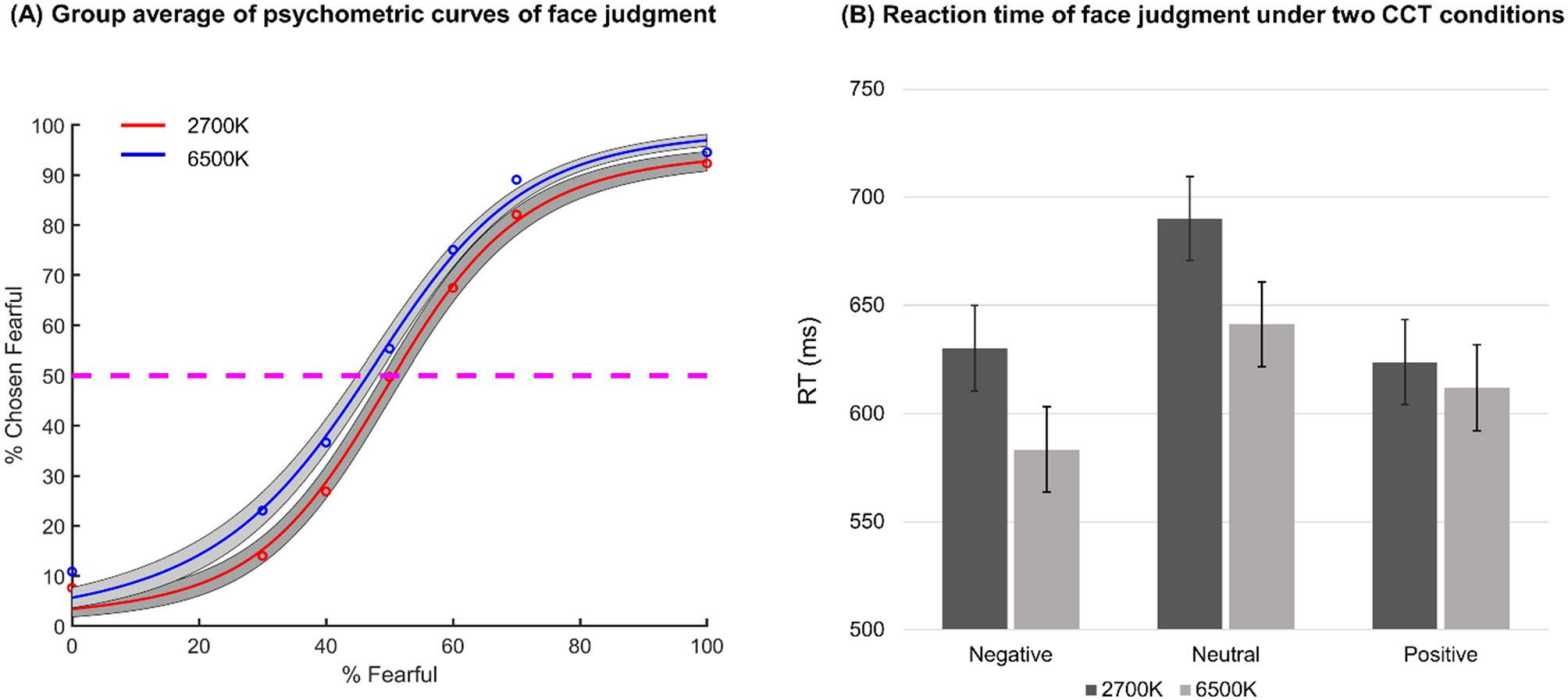
(**A**) Group average of psychometric curves showing the proportion of trials judged as fearful as a function of morph levels (ranging from 0% fearful (100% happy; on the left) to 100% fearful (0% happy; on the right)). Shaded area denotes ± SEM across subjects. (**B**) Face judgment reaction time of negative vs. neutral vs. positive faces under two CCT conditions. Whiskers represent standard errors.

Emotion Sensitivity Index (ESI), defined as the slope of the psychometric curves fitted by the above function, was used to investigate the sensitivity of emotional judgment at different morph levels. The results revealed a monotonically increasing relationship between the likelihood of identifying a face as fearful and the fearfulness in the morphed face for all participants (Fig. 3A). Participants under 2700 K (M = 1.47, SD = 0.32) and 6500 K (M = 1.42, SD = 0.29) light conditions had similar slopes (independent-samples t-Test, t (46) = 0.55, *p* = 0.59). Besides, the threshold, defined as the fearful chosen rate when the morph level was 50%, was also derived from the fitted curve for each participant under two CCT conditions. The results showed that the fearful chosen rate in 2700 K light condition (M = 48.63, SD = 17.51) was not significantly different from that in 6500 K light condition (M = 56.64, SD = 14.84) when the morph level was 50% (t (46) = − 1.71, *p* = 0.09).

For the response time (RT), LMM analysis revealed that the main effect of Valence was significant (F(2,570) = 92.73, *p* < 0.001); the post-hoc test showed that participants responded significantly slower to the neutral faces (EMM = 665.70, SE = 13.33) than to the positive faces (EMM = 617.88, SE = 13.50, *p* < 0.001) and the negative faces (EMM = 606.79, SE = 13.50, *p* < 0.001), while the reaction time for positive faces and negative faces was not significantly different (*p* > 0.05). In addition, the interaction effect between CCT and Valence was also significant (F (2,570) = 8.70, *p* < 0.001, R^2pseudo^ = 0.26). The post-hoc analysis indicated that participants’ reaction time for the negative, neutral, and positive faces did not significantly differ between 6500 K condition (negative: EMM ± SD = 583.45 ± 19.78; neutral: EMM ± SD = 641.25 ± 19.55, positive: EMM ± SD = 611.98 ± 19.78) and 2700 K condition (negative: EMM ± SD = 630.13 ± 19.67; neutral: EMM ± SD = 690.15 ± 19.45; positive: EMM ± SD = 623.79 ± 19.67) after Bonferroni correction (Fig. 3B). In addition, the results yielded no significant main or interaction effect of CCT and illuminance.

#### Emotional oddball task

**Figure 4:**
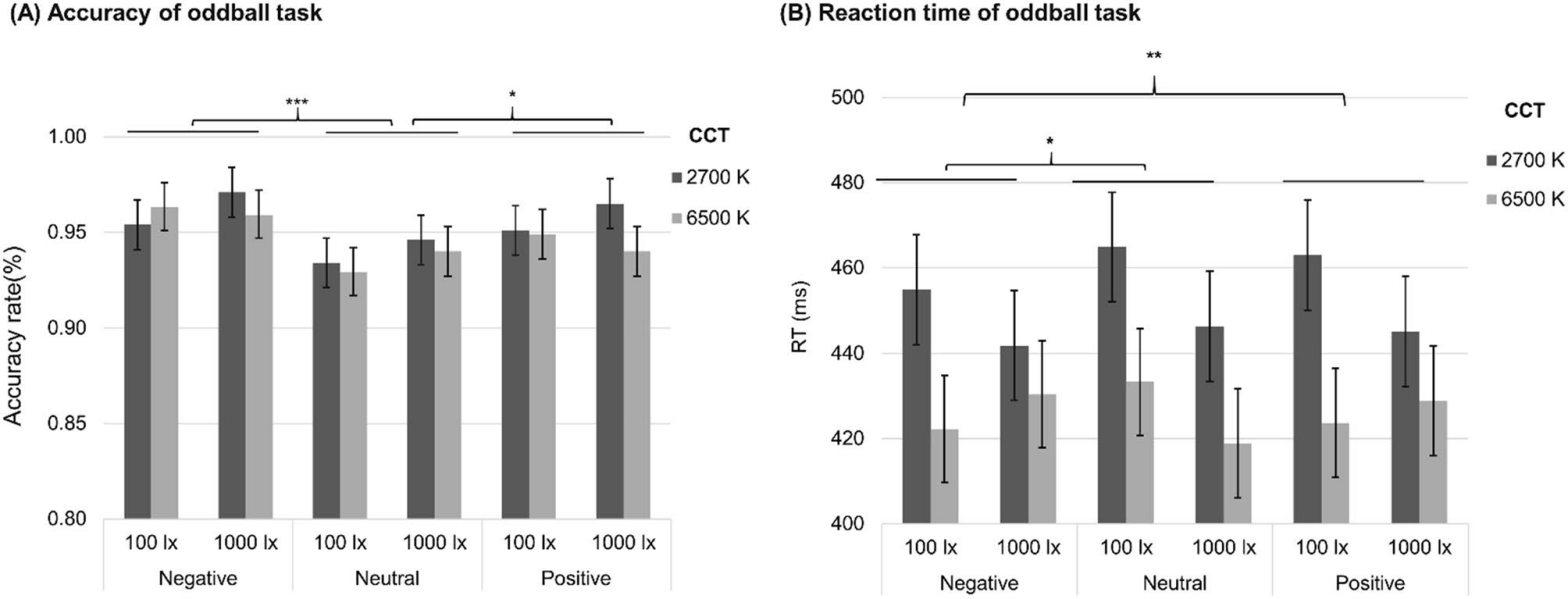
(**A**) Oddball accuracy of Negative vs. Neutral vs. Positive pictures under all light conditions. Whiskers represent standard errors. *p* < 0.05, ***p < 0.001. (**B**) Oddball reaction time of negative vs. neutral vs. positive pictures under all light conditions. Whiskers represent standard errors. *p < 0.05, **p < 0.01.

The LMM analysis of the accuracy rate in the oddball task revealed a similar response bias towards negative stimuli (F(2,186) = 11.96, *p* < 0.001, R^2pseudo^ = 0.13), with participants showing relatively higher response accuracy for the negative emotional stimulus (EMM = 0.96, SE = 0.01, *p* < 0.001) and positive emotional stimulus (EMM = 0.95, SE = 0.01, *p* = 0.02) compared to the neutral emotional stimulus (EMM = 0.94, SE = 0.01) (Fig. 4A). However, such response bias was not modulated by the illuminance and CCT manipulation (all *p *values > 0.05).

Neither the illuminance nor the CCT induced a main effect on the reaction time of emotional perception (illuminance: F(1,57) = 1.36, *p* = 0.25; CCT: F(1,50) = 3.73, *p* = 0.059), whereas the planed comparison indicated that participants’ reaction time tend to be shorter under 6500 K (EMM = 422.90, SE = 11.67) than 2700 K condition (EMM = 455.79, SE = 11.89, *p* = 0.059). The main effect of Valence was significant (F (2,186) = 7.13, *p* = 0.001, R^2pseudo^ = 0.08) (Fig. 4B), participants responded faster to the negative emotional stimulus (EMM = 434.35, SE = 8.25) than positive (EMM = 442.45, SE = 8.25, *p* = 0.002) and neutral emotional stimulus (EMM = 441.23, SE = 8.25, *p* = 0.01). Again, such difference was not susceptible to the influence from illuminance or CCT (*p *value > 0.05).

### Effects of lighting condition on subjective sleepiness and mood

LMM analysis showed that the lighting conditions had no significant main and interaction effects on subjective sleepiness and subjective mood (all *p* values > 0.05). LMM analysis for the light evaluation revealed a significant main effect of Illuminance on the subjective pleasantness (F(1,49) = 4.00, *p* = 0.05, R^2pseudo^ = 0.07), brightness (F(1,48) = 32.12, *p* < 0.001, R^2pseudo^ = 0.26) and excitement (F(1,95) = 17.11, *p* < 0.001, R^2pseudo^ = 0.18) of the light; participants evaluated the 100 lx light as more pleasant than the 1000 lx light, while they rated 1000 lx light as brighter and more exciting than the 100 lx light. Moreover, CCT elicited a significant effect on the perceived warmth (F (1,50) = 13.52, *p* = 0.001, R^2pseudo^ =  − 0.09), brightness (F (1,49) = 7.36, *p* = 0.009) of the light. Participants evaluated the 2700 K light as warmer than 6500 K light, but they perceived the 6500 K light as brighter than 2700 K light. In addition, no interaction effect between Illuminance and CCT was found (Table 3).

**Table 3:** Results for subjective measures (sleepiness, mood, evaluation of lighting conditions).

	2700 K		6500 K		Statistics									
	100 lx	1000 lx	100 lx	1000 lx	CCT			Illuminance			CCT*illuminance			R^2^
	EMM (SE)	EMM (SE)	EMM (SE)	EMM (SE)	F	*df*	*p*	F	*df*	*p*	F	*df*	*p*	
Sleepiness	4.18 (0.36)	4.06 (0.36)	4.21 (0.36)	4.4 (0.38)	0.19	(1,49)	0.67	0.01	(1,49)	0.91	0.31	(1,48)	0.58	0.07
Happiness	3.07 (0.14)	3.2 (0.14)	3.19 (0.14)	3.11 (0.14)	0.004	(1,48)	0.95	0.04	(1,48)	0.85	0.97	(1,47)	0.33	0.17
Sadness	2.37 (0.20)	2.12 (0.20)	2.45 (0.20)	2.67 (0.21)	1.69	(1,47)	0.20	0.01	(1,46)	0.91	2.37	(1,45)	0.13	0.06
Pleasantness	3.41 (0.19)	3.09 (0.19)	3.69 (0.19)	3.27 (0.20)	1.28	(1,48)	0.26	4.00	(1,49)	**0.05**	0.07	(1,48)	0.79	0.07
Comfort	3.29 (0.23)	3.06 (0.23)	3.52 (0.23)	3.13 (0.24)	0.34	(1,50)	0.57	2.45	(1,49)	0.12	0.16	(1,49)	0.69	0.10
Disturb	3.61 (0.21)	3.39 (0.21)	3.51 (0.21)	3.25 (0.22)	0.27	(1,50)	0.61	1.34	(1,50)	0.25	0.01	(1,49)	0.93	0.04
Warmth	3.63 (0.20)	3.73 (0.20)	2.98 (0.20)	2.82 (0.21)	13.52	(1,50)	**0.001**	0.02	(1,49)	0.88	0.45	(1,48)	0.50	− 0.09
Preference	3.09 (0.19)	2.79 (0.19)	3.01 (0.19)	3.02 (0.20)	0.14	(1,50)	0.71	0.59	(1,50)	0.45	0.67	(1,49)	0.42	0.02
Brightness	2.62 (0.21)	3.64 (0.21)	3.03 (0.21)	4.43 (0.22)	7.36	(1,49)	**0.009**	32.12	(1,48)	**0.00**	0.80	(1,48)	0.38	0.26
Excitement	2.35 (0.22)	3.23 (0.22)	2.74 (0.22)	3.67 (0.23)	3.34	(1,95)	0.07	17.11	(1,95)	**0.00**	0.01	(1,95)	0.93	0.18
Effect on work	3.02 (0.17)	2.83 (0.17)	3.29 (0.17)	3.16 (0.18)	2.38	(1,50)	0.13	1.15	(1,50)	0.29	0.03	(1,49)	0.86	0.01
Effect on mood	3.25 (0.14)	3.21 (0.14)	3.19 (0.14)	3.1 (0.15)	0.31	(1,48)	0.58	0.27	(1,48)	0.61	0.03	(1,47)	0.87	− 0.09

*EMM* estimated marginal means, *SE* standard errors.

A significant difference is indicated by bold labels.

## Discussion

Although the antidepressant effect of light therapy and the acute effects of short-term light exposure on subjective affective state have been widely investigated, few studies have investigated the acute effects of indoor light on pure emotional perception. The current study was conducted to explore the individual and interaction regulation of illuminance and CCT of inddor light on explicit and implicit emotion perception tasks with controlling the prior affective and vigliance state. The results mainly revealed that the lower CCT decreased the negative response bias during emotional face judgment. Nevertheless, no such modulation effects of CCT were found on attentional negativity bias as assessed with the emotional oddball task. Reaction speed to emotional faces and pictures was not significantly affected with light manipulation. Moreover, the results did not reveal any significant effects of light manipulations on subjective momentary alertness and mood. These findings contributed to the current studies suggesting a type of task-dependent modulation effect of CCT in emotional perception and the differential role of CCT and the illuminance of indoor light in regulating emotion perception.

For the explicit emotional perception, the CCT manipulation significantly regulated individuals' negative response bias, such that participants perceived the same set of faces as less fearful in 2700 K light than in 6500 K light in the emotional face judgment task. Furthermore, it is notable that reaction time may also have to do with this negativity bias, as a previous study found that there was a significant correlation between reaction time and valence bias, that is, the faster participants responded to ambiguous faces, the more likely they were to judge them as negative^72^. We thus conducted an additional statistical analysis of the reaction time at seven fearful levels, and the results showed that the RT of different fearful levels under the two CCT conditions were not significant (*p* = 0.24), suggesting there was no contribution of the correlation between the reaction speed and negativity bias in the current study.

The light-rendered emotional scene could partly explain these findings since facial expression recognition was proved to be susceptible to scene information (a picture of an emotional scene that presents before or simultaneously as the face stimulus present)^73–75^. To be specific, the room with warm-white light (less than 3500 K) was inclined to be perceived as a more positive (i.e., pleasant, attractive, and relaxed) atmosphere than a room with cool-white light (with relatively high CCT). In contrast, light with high CCT (equal to or greater than 5000 K) was perceived as a more negative atmosphere than light with low CCT^76–79^. The findings from the study by Lee et al.^80^ also revealed that individuals' perceptual thresholds for a morphed face continuum(neutral (0%) to fearful (100%) in 10% increments) was influenced by scene information, with ambiguous fearful facial expressions being more efficiently categorized as fearful when they were embedded in a negative scene relative to a neutral or positive one. Thus, in the current study, the ambient light presented a different color appearance due to its different CCT, which also led to the occurrence of scene effects.

Another possible explanation for the low CCT induced regulation of attenuated negative emotional bias is that the low ambient CCT induced warm perception of ambient light might prime participants to perceive neutral faces as more positive. One recent study by Gu et al.^81^ reported that individuals were more likely to recognize neutral faces as happy on a warm-colored background than a cool-colored background. These findings could be explained by the embodied cognition theory, suggesting the perception of the physical environment (such as cold and warm, bright and dark) will trigger the activation of the higher-level psychological concepts (such as good and bad)^82,83^. For instance, the previous studies reported that environmentally induced warm conditions (touching warm beverages/pack or placing participants in warm ambient conditions) were associated with more positive behaviors^84,85^.

Contrary to explicit emotional perception, the implicit emotion perception was not significantly regulated by the manipulation of light level and CCT. However, the current findings revealed a stronger attentional bias to the emotional pictures, with higher accuracy and faster reaction speed for positive and negative pictures than neutral pictures, regardless of the light manipulations. These findings were comparable to the study by Huang and Luo^86^, which revealed an emotional effect, as evidenced by mean reaction time was as shorter for emotional stimuli than neutral stimuli. Differentiating versus explicit emotional processing, this performance improvement in emotional stimulus was independent of CCT and illuminance, suggesting that CCT did not moderate the attentional bias in the implicit task.

Regarding the task-dependent regulation (only manifested in the explicit emotion task) of lighting on emotional perception in the current study, this may be due to the differences in the dominant processes of these two tasks. The judgment of the face inclined to be perceptual processing via "top-down" influences^87^, while the response to the emotional pictures in the oddball task involved more "bottom-up" attention^88^. As stated in the introduction, bright light and light of short wavelength could modulate neural activities in both top-down and bottom-up attentional pathways^89^. Different from high-intensity polychromatic light and monochromatic blue light, the polychromatic light with a relatively low blue spectrum may only have a more significant regulatory effect on a particular neural pathway (i.e., the top-down processes). Nevertheless, as the behavioral measurement approach was employed in the current, we had no conclusive findings of this explanation.

In addition to explicit and implicit emotion perception, the current study also investigated the acute effects of light manipulation on momentary mood and sleepiness. The findings revealed no statistically significant individual or interacted regulation of high illuminance and CCT level on subjective sleepiness and mood. This finding is in line with several studies in which participants' subjective sleepiness remained unaffected after 50 min exposure to 1000 lx vs. 200 lx light^25,34^ as well to 6000 K vs. 2700 K light^30^. Furthermore, the null effect of CCT level on sleepiness in the current study may be explained by the fact that the short exposure duration and medium–high CCT level were insufficient to produce an alerting effect. There was evidence that the alerting effects of a higher CCT level were occasionally reported when more extended exposure periods (several weeks) or extremely high CCT levels (17,000 K vs. 4000 K)^29,90^ was employed.

Similarly, the subjective mood remained insensitive to the illuminance and CCT manipulation in the current study, reflecting the results of the previous studies^28,33,34^. A possible explanation might be that the two-item scale employed in the current study might not be sensitive to detecting tiny changes in mood. Other researchers reported significant effects of illuminance or CCT of daytime light on the mood that was assessed with multiple items questionaires^25,39^. Moreover, participants in the current study had a relatively agreeable emotional state at baseline (EMM = 3.69 for happy and EMM = 2.00 for sad on the five-point items), such that lead a limited room for improvement of mood with light manipulation. In contrast to the effects on alertness and mood, the current findings demonstrate a more consistent picture regarding light's effects on subjective evaluation of experimental light. The light of 1000 lx and light of 6500 K were both perceived as brighter and more exciting; the light of 1000 lx was rated as more pleasant than the light of 100 lx, and the light of 2700 K was rated as warmer than the light of 6500 K.

Some limitations of the current study must be considered before generalizing the experimental results. First, the light exposure duration in the current study was relatively short (only about 30 min), and its modulatory effect on mood and negativity bias may not be fully manifested. Secondly, the effect of time of day was not considered in this study due to the limited number of samples, which was established as a moderator of the diurnal NIF effects of light on physiological and psychological activities^30^. Thirdly, the non-nonclinical sample employed might limit the transfer of the current findings into the clinical field. Future studies should conduct a randomized controlled trial on the effect of light on emotional processing among individuals diagnosed with seasonal affective disorder.

## Conclusion

In conclusion, no significant main or interaction effects of illuminance and CCT on subjective sleepiness and mood were found in the current study. The findings demonstrated that low CCT decreased individuals' negative response bias, labeling ambiguous faces as less fearful during explicit emotional judgment independent of illuminance. In comparison, no significant regulating effect of light (illuminance and CCT) on the attentional negativity bias in the implicit emotional oddball task was revealed. These findings contribute to the current studies suggesting a type of task-dependent modulation effect of CCT in emotional perception and the differential role of CCT and the illuminance of indoor light in regulating emotion perception.

## Data Availability

The datasets generated during and/or analysed during the current study are available from the corresponding author on reasonable request.
